# The U.S. Global Gag Rule in Ethiopia: a foreign policy challenging domestic sexual and reproductive health and rights gains

**DOI:** 10.1186/s12978-022-01329-6

**Published:** 2022-06-13

**Authors:** Jamie M. Vernaelde

**Affiliations:** 1 PAI, 1300 19th Street NW, Suite 200, Washington, DC 20036 USA; 2 Ipas, P.O. Box 9990, Chapel Hill, NC 27515 USA

**Keywords:** Ethiopia, Global Gag Rule, Sexual and reproductive health, Abortion, Foreign policy, Family planning, Maternal mortality, United States, Trump

## Abstract

**Background:**

Ethiopia’s government and civil society have driven crosscutting initiatives in the last 15 years to improve sexual and reproductive health outcomes, including passing a 2005 abortion law that facilitated reduced rates of maternal death due to unsafe abortion. However, both the government and nongovernmental organizations have relied on external funding for sexual and reproductive health and rights, particularly from the U.S. government, which has been Ethiopia’s largest global health donor. This article explores how the implementation and expansion of the 2017–2021 U.S. foreign policy “Protecting Life in Global Health Assistance,” also known as the Global Gag Rule—which attached itself to a nongovernmental organization’s funding—impacted sexual and reproductive health and rights, including safe abortion care, in Ethiopia.

**Methods:**

This article is based on research conducted by PAI staff in Ethiopia in 2018 with follow-up in 2019. PAI held in-depth semistructured interviews with representatives of 30 organizations in Ethiopia’s capital, Addis Ababa. Among these groups were U.S.-based and non-U.S. nongovernmental organizations, including community-based organizations, non-U.S. government donors, and Ethiopian government officials.

**Results:**

Nongovernmental organizations have been essential to sexual and reproductive health service provision and advocacy in Ethiopia. Because of the sector’s reliance on U.S. global health assistance, these organizations; their activities; and, consequently, the wider health system were negatively impacted by the Global Gag Rule. Certain vulnerable groups, particularly adolescents and youth, have traditionally relied on the private sector for sexual and reproductive health services. PAI’s research demonstrates that U.S. policy disrupted activities and service delivery, threatened the closure of private clinics, stalled mobile outreach, and impacted safe abortion training of health personnel. Additionally, the Global Gag Rule dismantled partnerships, affected non-U.S. government donors’ investments, and caused confusion that limited activities permissible under the policy.

**Conclusions:**

The Trump administration’s Global Gag Rule forced non-U.S. organizations to choose between providing comprehensive care or losing U.S. global health assistance, ultimately impacting populations in need of services. Ethiopia provides a clear example of how the Global Gag Rule can threaten a country’s domestic health agenda by targeting nongovernmental organizations that are vital to health service delivery and safe abortion care.

## Plain language summary

In 2005, Ethiopia’s government, health advocates, and service providers secured a more liberal abortion law that has been instrumental in reducing maternal deaths due to unsafe abortion. However, these fragile gains are vulnerable because the in-country sexual and reproductive health sector has relied heavily on external funding. The U.S. government has been an important development partner to Ethiopia for global health and, in particular, reproductive health and family planning. As a result, the Global Gag Rule, reimposed and expanded by the Trump administration in 2017, impacted sexual and reproductive health and rights in Ethiopia. U.S. agencies and departments attached this policy to U.S. global health assistance for nongovernmental organizations that were critical partners in service delivery and advocacy. In a country like Ethiopia with a progressive environment for sexual and reproductive health, including safe abortion care, it remains important to examine the impacts of the Global Gag Rule, despite being rescinded in January 2021 by the Biden administration, as the policy has still not yet been permanently repealed and could be reinstated by future administrations.

PAI’s research sought to document the impacts of the expanded Global Gag Rule on sexual and reproductive health in Ethiopia, with a focus on safe abortion care. Study participants reported that nongovernmental organizations serving rural populations, adolescents and youth, sex workers, and people living with HIV/AIDS were impacted by the loss of U.S. government funding due to noncompliance with the policy. Organizations that chose to comply with the policy were forced to stop critical activities like training on safe abortion care. Outcomes also included self-censorship out of fear of the policy and dismantled partnerships between compliant and noncompliant organizations.

## Background

The Ethiopian government has driven crosscutting initiatives to improve access to sexual and reproductive health and rights (SRHR) information, education, and services for its population of 102 million people. In the last 15 years, this course of action included passing a progressive abortion law in 2005 and adding subsequent implementation guidelines that allow for pregnancy termination under certain circumstances [1]. In addition to its legality in cases of life endangerment, rape, or incest, abortion is legal if the pregnant person—owing to physical or mental reasons, including being a minor—is unprepared to raise a child [1]. Evidence suggests that maternal mortality, historically high in Ethiopia and particularly associated with unsafe abortion, declined with increased uptake in family planning and improved access to legal, comprehensive abortion care [2].

In this environment, demand for family planning steadily increased since the early 2000s. Modern contraceptive use among married Ethiopian women climbed from 6% in 2000 to 35% in 2016 [3]. According to the Federal Ministry of Health, this success was due to increased access to facilities, improved contraceptive supply chain management, and the decentralization of family planning services through community health extension workers, as well as support from partnerships with nongovernmental organizations (NGOs) [4]. Currently, contraception is free throughout the country, and the Federal Ministry of Health provides contraceptive supplies to the private sector. As of 2019 data, contraceptive uptake was trending positively toward the national goal of increasing contraceptive prevalence among 15- to 19-year-old women to 40% and 20- to 24-year-old women to 43% by 2020 [5]. The government further committed to reducing the unmet need for those two age groups to 10% overall [5]. As part of its efforts to increase contraceptive uptake among key populations, including the 50% under age 20, the government prioritized initiatives targeting the unmet need of adolescents; in its national guidelines on family planning services, Ethiopia’s government recognized the unique sexual and reproductive health (SRH) challenges of young people and developed an adolescent and youth strategy specific to reproductive health [6, 7].

Despite this progress, ensuring the continuity of reproductive health supplies provision and distribution to the last mile continue to pose challenges for Ethiopia’s government. Fertility rates remain high and contraceptive use varies significantly across the country, with 10% more women in urban settings using a modern contraceptive method than those living in rural areas [8, 9]. Among rural populations, which comprise 80% of the country, contraceptive uptake and demand generation are limited [10]. To complement the work of the public sector, the government has depended upon the private sector and local and international NGOs to deliver a range of SRH services and contraceptives [6]. These organizations also have provided training for public health workers, including midwives; supported contraceptive supply chain management; and coordinated on SRHR policy development and implementation. Though most people access contraceptives through government facilities, an estimated 20% use the private sector and 40% of pregnant people still prefer to seek safe abortion care through private facilities [2, 11]. Although safe abortion care is accessible for many people in the country—53% of induced abortions in 2014 were performed in public and private health facilities—unsafe abortions continue [12]. It is estimated that 40% of abortions performed outside of health facilities result in serious complications [12].

As Ethiopia was making sexual and reproductive health gains, President Trump reinstated the Mexico City Policy in January 2017. Formally renamed as “Protecting Life in Global Health Assistance,” the policy is known by its critics as the Global Gag Rule (GGR). The Trump administration extensively expanded the GGR beyond versions of the policy, under past Republican presidents, that only applied to family planning and reproductive health (FP/RH) assistance. With the Trump administration, U.S. agencies and departments attached the GGR to all U.S. global health assistance, impacting funding for FP/RH as well as maternal and child health, HIV/AIDS prevention and treatment, malaria, nutrition, and even certain water and sanitation programs, among others. The GGR effectively prohibited non-U.S. NGOs that received U.S. global health assistance from using their private, non-U.S. funds to provide comprehensive, safe abortion services for reasons other than life endangerment, rape, or incest; to offer information or referrals for abortions; or to advocate for the legalization of safe abortion services beyond the exceptions for life endangerment, rape, or incest. While the GGR was rescinded by executive order by the Biden administration in January 2021, the policy has still not yet been permanently repealed and could be reinstated by future administrations.

The Trump administration’s GGR extended further than preexisting legal restrictions on the use of U.S. government funds for safe abortion care—specifically the Helms Amendment, which has been in place since 1973—to limit what an organization can do with its private, non-U.S. government funding [13]. Following the GGR’s initial expansion to all U.S. global health assistance under the Trump administration in 2017, a new interpretation of the existing language implementing the policy was released in March 2019 by then-U.S. Secretary of State Mike Pompeo. The subsequent interpretation effectively prohibited a GGR-compliant non-U.S. NGO from using its non-U.S. government assistance to support any kind of health or development work of a non-U.S. partner that received no U.S. government global health assistance if that partner separately engaged in abortion-related work with its own funding [14]. This meant that, in practice, for an organization that received U.S. global health assistance, the GGR effectively attached to all its separate funding—beyond global health activities—from any of its other non-U.S. donors.

The Federal Ministry of Health and NGOs in Ethiopia have been vulnerable to these changes in U.S. foreign policy because of their reliance on external donor funding for SRH activities and supplies. The U.S. government has been the single largest global health donor to Ethiopia, with nearly USD 250 million obligated in fiscal year 2017 [15]. After the United Kingdom—which allocated GBP 90 million over four years to Ethiopia in 2017—the United States was the second-largest donor for family planning specifically, followed by the Netherlands, the United Nations Population Fund, and other key bilateral donors and foundations [16, 17].

U.S. global health assistance flows from multiple U.S. government sources, including the U.S. Agency for International Development (USAID), the U.S. Centers for Disease Control and Prevention (CDC), and the U.S. Department of State, among others. In partnership with the government and civil society in Ethiopia, USAID has had a key role in improving SRH outcomes, ensuring access and availability of modern contraceptives, and increasing access to high-quality family planning services [18]. In 2017, USAID obligated USD 125 million for FP/RH, HIV/AIDS prevention and care, and maternal and child health programs in Ethiopia [15]. More than 77% of that funding went to 10 not-for-profit NGO prime recipients that have significant networks of partner organizations in-country, some with as many as 20 subrecipients on a given grant [15, 19]. These organizational figures were not comprehensive of all U.S. global health assistance to Ethiopia; they excluded funds from agencies like the U.S. President’s Emergency Plan for AIDS Relief (PEPFAR) and the CDC. The CDC and PEPFAR have different reporting periods from USAID as well as additional numbers of prime recipients and subrecipients. All prime recipients and subrecipients of U.S. global health assistance must comply with language in their grant agreements, which have unique implications for SRHR due to the flux in U.S. administrations and their policies, such as the GGR.

PAI documented the impacts of previous iterations of the GGR on SRHR globally, with an emphasis on contraceptive supplies and maternal deaths due to unsafe abortion [20–22]. With the Trump administration’s extensive expansions of the GGR, PAI renewed its fact-finding documentation between 2018 and 2019 to understand preliminary effects of the then-new policy across different countries receiving U.S. global health assistance, including Ethiopia. This documentation coincided with peer-reviewed research and gray literature on the impact of previous iterations of the GGR on SRH services and overall health outcomes [23]. Several quantitative studies that focused on the Bush administration’s implementation of the GGR found associations between the policy and a reduction in modern contraceptive use as well as an increase in abortions in sub-Saharan Africa and Latin America and the Caribbean [24–26]. Under both the 2001–2009 Bush GGR and the multiple 2017–2021 Trump expansions, research documented reductions in service delivery and availability impacting not just FP/RH but also maternal and child health, HIV/AIDS, and tuberculosis, among other areas of global health [27–29]. Beyond substantiating direct service delivery impacts, research on the GGR also documented what activists call the “chilling effect” of the policy, encompassing both an over-implementation of the GGR in activities and programs as well as self-censorship with advocacy and in-country partnerships [28, 30, 31].

In the case of Ethiopia, when the GGR was in effect and limited to U.S. FP/RH funding during the Bush administration, PAI documented severe financial damage to certain NGOs due to noncompliance with the policy as well as the loss of USAID-donated contraceptives, which then worsened Ethiopia’s supply shortage [32]. With the Trump administration’s expansion of the policy to all U.S. global health assistance, many more organizations in Ethiopia receiving U.S. funding had to follow its restrictions, even if their projects were supported by non-U.S. government donors [33]. Based on PAI’s research from 2018 to 2019, this article examines the extent to which the early roll out of the expanded GGR under the Trump administration disrupted NGO activities and progress in comprehensive sexual and reproductive health, including safe abortion care, in Ethiopia.

## Methods

This article is based on an exploratory case study documenting the impacts of the Trump administration’s 2017 expanded GGR on SRHR in Ethiopia conducted by two PAI staff members [34]. Research was executed through semistructured interviews with NGO and community-based organization (CBO) directors and program managers, FP/RH or global health focal points from bilateral donor governments, and other donor agencies as well as relevant representatives from the Federal Ministry of Health and local health administration from 30 organizations in Addis Ababa, Ethiopia. Interviews were conducted in February 2018 with follow-up in December 2019. Because of a national state of emergency declared in Ethiopia at the time of research in 2018, PAI staff were not able to travel outside of Addis Ababa and the follow-up trip limited additional interviews.

PAI purposively selected the organizations to reflect a range of exposure to the GGR and included U.S. and non-U.S. NGOs providing SRH services or engaging in SRHR advocacy—both direct prime recipients and subrecipients of U.S. global health assistance. Not all participants interviewed during the initial visit in February 2018 were interviewed in the December 2019 follow-up, though there was significant overlap among the prominent NGOs implementing U.S. global health assistance programs. NGOs receiving U.S. global health assistance were selected from the U.S. government’s foreign assistance open data platform [15]. Additional interviewees were identified through a search of SRHR actors operating in Ethiopia; at the February 2018 First Annual Scientific Reproductive Heath Conference in Addis Ababa; and through snowball sampling to connect with subrecipients, particularly CBOs [4].

Study participants were from U.S.-based and non-U.S. NGOs, including those that chose to comply with the GGR and those that did not. The U.S.-based NGOs provided information on their subrecipients—those that chose to comply with the GGR—and, where needed, the methods U.S. NGOs used to enforce GGR compliance with these subrecipient partners. Non-U.S. NGO participants represented organizations that were prime recipients of U.S. global health assistance, subrecipients, or both. Table 1 provides a breakdown of the types of organizations represented in interviews by category. For confidentiality, identifying information has been omitted, including an organization’s decision regarding compliance with the GGR. Not all organizations contacted by PAI agreed to be interviewed, including non-U.S. NGOs and CBOs complying with the policy as prime recipients and subrecipients of U.S. global health assistance. Some of those who declined to participate clearly stated their belief that speaking about the GGR was perceived to be impermissible under the policy’s restrictions.

**Table 1:** Organizational representation by type

Bilateral donor governments and other donor agencies	6
Directorates within the Federal Ministry of Health	2
Non-U.S. NGOs (including other international NGOs as well as Ethiopian NGOs and CBOs)	10
U.S.-based NGOs	12

The study interviews were designed to determine participants’ exposure to the GGR and the resulting impacts as well as how the policy might have wider implications for Ethiopia’s health system. PAI developed a semistructured interview guide in English for each of the following participant categories: U.S.-based NGOs, non-U.S. prime NGOs or subrecipients, and government and non-U.S. government donors. Questions for NGOs were organized based on whether they received U.S. funding, whether they were complying with the GGR, and how the decision to comply would affect their organizational activities and beneficiaries. Questions were also designed to inform PAI’s understanding of the context of U.S. funding and other donor funding for SRHR in Ethiopia—an understanding critical for gauging the policy’s impact on non-U.S. government donors. For interviews with officials and members of the Ethiopian government, additional questions focused on the role of civil society in service provision, the implications of losing U.S. funds for members of civil society, and participants’ observed impacts of the GGR. This methodological approach was particularly useful in identifying evidence of the varied impacts of the policy on the Ethiopian health system.

All interviews lasted between 60 and 90 minutes and were conducted in English. With all stakeholders, the purpose of the interview and the way the information would be used were discussed along with the interview’s voluntary and confidential nature. All names of individuals and organizations were withheld unless consent was given to provide identifying information. As part of the interviews, technical assistance was provided on the GGR in the form of an explanation of the expanded policy under the Trump administration, followed by a discussion on how it differed from prior iterations of the policy. The technical assistance was followed by a discussion, distribution of resources for participants to better understand the policy, and an opportunity to have questions answered. In all interviews, PAI staff stressed the importance of a clear understanding of the expanded GGR and the need, especially for NGO recipients of U.S. global health funding, to seek clarification from their funding source—the U.S. government or the prime recipient. Detailed interview notes were coded manually and analyzed by type of organization with descriptive codes to cover key themes. To supplement interview data, PAI staff consulted gray literature in the form of organizational and government reports, program evaluations, and other documents.

## Results

### Impact on SRH services and safe abortion care

Study participants from across the different types of organizations interviewed revealed negative effects of the policy on SRH program activities, service delivery, and safe abortion care. The two largest SRH service delivery NGOs in Ethiopia, Marie Stopes Ethiopia and the Family Guidance Association of Ethiopia (FGAE), are non-U.S. organizations that chose to not comply with the GGR. The resulting loss of U.S. funding for these organizations threatened the closure of clinics, and several participants—including government and donor representatives as well as the organizations themselves—reported delayed or stalled outreach for rural populations that were unable to receive their contraceptive methods of choice.

FGAE, an International Planned Parenthood Federation member association, has worked in Ethiopia for more than a half century providing SRH services and contraceptives through its 47 clinics and support to 350 other health facilities [35]. In response to FGAE’s decision to not comply with the GGR, the CDC withdrew a five-year grant awarded in 2017 that would have averaged USD 2 million per year [4]. According to participants, if not for short-term replacement funding from the government of the Netherlands, the forfeiture of CDC funds would have resulted in the closure of 10 confidential, sex worker–friendly clinics and compromised 21 additional clinics where the CDC partially supported integrated HIV/AIDS services. Without these clinics, more than 15,000 female sex workers and almost 790,000 women, men, and young people were at risk of losing access to lifesaving care. As a result of its noncompliance, FGAE was obligated to return some U.S. assets and equipment received over the last seven years of U.S. government support. It also lost CDC training on planned antiretroviral therapies for its HIV/AIDS work. Additionally, because of the GGR, a U.S.-based prime recipient of U.S. global health assistance ruptured its partnership with FGAE, resulting in the loss of in-kind contraceptives valued at USD 800,000 and trainings for FGAE staff on cervical cancer screenings. FGAE reported having to allocate funds from each of its other programs and having to negotiate to buy more contraceptives cheaply from other sources to cover the contraceptive loss. The Federal Ministry of Health stepped in to support FGAE, though at the expense of other health priority areas, like refugee support and nutrition for communities displaced by drought and conflict [4].

In 2018, after deciding to not comply with the GGR, Marie Stopes Ethiopia closed out its USAID program that complemented the contraceptive method mix and choice available in the public sector. Before the closure, the organization’s 13 mobile outreach teams had provided contraceptive options to underserved, rural populations [36]. As one participant explained:

Hard-to-reach areas require double or triple effort. You may need to drive 100 kilometers to reach one woman, but she has the right to family planning.—U.S.-based NGO representative, 2018

With the loss of USAID funds, Marie Stopes Ethiopia’s provision of permanent contraceptive methods—specifically, vasectomies and tubal ligations—ended. In one district, a local health office that had worked with Marie Stopes Ethiopia described the impact of this loss on counseling and services: community members continued to request permanent methods, but there was no available provider. Through a combination of domestic funding and support from the United Kingdom, eight Marie Stopes Ethiopia mobile teams continued to operate, though they redeployed and no longer offered permanent, surgical contraceptive methods because those methods are more time consuming and complex to provide.

If MSI wasn’t here it would be very tough, especially to reduce maternal mortality. Mothers now use family planning. And mothers would die if MSI were not here. There would be more unwanted pregnancies.—Government representative, 2019

These GGR impacts on Ethiopia’s largest SRH service delivery NGOs restricted access for vulnerable populations, specifically, young people and sex workers. Multiple participants, including from the Federal Ministry of Health, acknowledged that certain groups prefer to seek out services in the private and NGO sectors over the public sector to ensure privacy and avoid perceived stigma.

Private providers are the key for service delivery to vulnerable populations, including youth and sex workers who rely on them for integrated services, including HIV/AIDS prevention and treatment, family planning, and in some cases, safe abortion care.—U.S.-based NGO representative, 2018

This reliance on private providers for safe abortion care was identified to be particularly significant for adolescents and youth.

When we talk about abortion, it is about the spectrum of care and the client’s rights. Young people go to MSI and FGAE for privacy. They don’t want to queue with their aunts for the public health facilities. Abortion is still stigmatized. [Public] providers still don’t want to provide it.—Non-U.S. NGO representative, 2018

Two youth-focused CBOs shared that their decision to not comply with the GGR was because of the need to continue providing safe abortion care information and education to young people.

It’s about knowing the need, and it’s a major need for youth as part of sexual and reproductive health.—U.S. NGO representative, 2018

In addition to describing these direct losses for noncompliant NGOs, study participants reported that the GGR created regional disparities in comprehensive SRH service availability. Large U.S.-based NGOs that were prime recipients of U.S. global health assistance had extensive reach in Ethiopia and worked in different regions with a variety of subrecipients. For example, a five-year project led by a U.S.-based NGO with non-U.S. NGO subrecipients supported public health facilities in 75% of the country, or 500 of its 800 districts. A non-U.S. NGO subrecipient on that project worked with nearly half of the 12,500 midwives throughout Ethiopia. As a result of compliance with the GGR, the NGO no longer offered trainings to midwives on safe abortion care—despite the fact that the GGR was not attached to bilateral funding for the Ethiopian government and midwives are government workers. Four other study participants from both compliant and noncompliant U.S.-based and non-U.S. NGOs expressed concerns that, given this organization’s reach throughout the country, the GGR’s effects on its safe abortion care trainings would impact postpartum care and safe abortion care services in Ethiopia more broadly.

For maternal and child health services and adolescent and youth health services, the nearest provision of service is midwives, and this [the GGR] creates gaps in services.—Non-U.S. NGO representative, 2019

Two U.S. organizations that continued to work on comprehensive abortion care attempted to address the safe abortion care gap in the U.S.-funded districts but reported lacking the necessary funding to accomplish this in all locations.

Comprehensive abortion care in those areas is the biggest gap in public health facilities.—Non-U.S. NGO representative, 2019

### Impact on partnerships

The interaction of the GGR, including the loss of service providers who declined to comply with the policy, and Ethiopia’s liberalized abortion law created a complex environment for SRHR NGOs and their donors. Study participants from U.S.-based NGOs, non-U.S. NGOs, and CBOs as well as government and donor agencies all mentioned that the GGR was negatively impacting partnerships and their ability to advance SRHR projects. In some cases, prime U.S.-based and non-U.S. NGOs receiving U.S. global health assistance had to sever relationships with long-standing non-U.S. NGO partners that declined to comply with the policy.

The effect of the GGR is beyond its financial and material implications. It’s about disrupting partnerships, disrupting integrated services, efforts to promote leveraging, efforts to coordinate resources among partners.—Non-U.S. NGO representative, 2018

Three CBO representatives that participated in the study reported choosing to not comply with the GGR in order to continue working on safe abortion care—foregoing funded partnerships with compliant prime U.S. and non-U.S. organizations. This rupture in partnerships undermined the quality of comprehensive care delivered by compliant organizations that were no longer able to work with noncompliant CBOs. Non-U.S. NGOs and local CBOs offer skills and technical capacity to reach rural, adolescent and youth, and marginalized populations. One prime organization that was compliant with the GGR had to dissolve partnerships in 2018 with several non-U.S. NGO partners that chose to not comply with the GGR, including FGAE. The compliant organization recognized that the subsequent reorganizing of the intended project due to the GGR affected its quality and efficacy.

FGAE is more networked. They’ve been around for years. Working with them you know what you’re doing is sustainable. They work all across the country and others [organizations] don’t. FGAE also does demand creation and people go to them for different services, so they’re highly visible.—Multilateral agency representative, 2018

Although several non-U.S. government donors committed to support NGOs that experienced funding losses because of the GGR, the policy also directly impacted their investments and ability to partner with U.S.-funded NGOs. For example, a Dutch-funded project of USD 9 million over four years for comprehensive abortion care was delayed because the lead organization complied with the GGR and could no longer complete the work. After this role was transferred to another NGO partner, the project was severely disrupted, including its delivery of family planning and safe abortion care services. In the case of the United Kingdom, the Department for International Development dedicated GBP 90 million over four years for work in Ethiopia with the Federal Ministry of Health to provide modern contraceptive methods. The primary NGO partner complied with the GGR and, to adhere to its restrictions, stopped working with other non-U.S. NGOs on the project. There was also an attempt to isolate safe abortion care, segregating those services from the other project activities, which caused the program to halt for nine months. Ultimately, the policy impacted the ability of non-U.S. donors to invest long term in key, GGR compliant non-U.S. NGOs and local CBOs, affecting sustainability of activities and the non-U.S. NGOs themselves.

Local organizations that have signed the Global Gag Rule cannot do referrals [for abortion]. They cannot get other donor funding. We cannot work with the local organizations, and it forces us to work with the internationals [NGOs]. Local ownership and capacity building are lost. If there are another five years of this, we lose grassroots family planning. It’s not just the money, it’s the technical support, the help with other donors.—Non-U.S. donor representative, 2019

### Impact of over-implementation and self-censorship

Participant responses during the study suggested that the GGR was being over-implemented in Ethiopia, particularly through enforcement by large U.S.-based organizations on their non-U.S. NGO subrecipients. At least three U.S.-based, compliant prime organizations that received the majority of their funds from the U.S. government expressed concerns about how the GGR would negatively impact Ethiopia’s progress with reducing maternal mortality due to unsafe abortion. These NGOs specifically cited fear of the policy’s effect on post-abortion care, despite its permissibility under the GGR. This form of over-implementation of the policy’s restrictions is often referred to as its “chilling effect.”

The chilling [effect] is beyond the funding loss and loss of partnerships. There is fear around abortion. When we talk with health managers, they don’t want to talk about abortion. Before they were integrating safe abortion care into their services. The chilling effect is fear from organizations. If they [do not] talk or communicate about abortion, [or build] the capacity of the government with health extension leads or lower-level cadres, the whole progress may collapse in the long run.—Non-U.S. NGO representative, 2018

In addition to describing halting permissible activities, study participants reported over-implementation in the form of self-censorship. The refusal of certain compliant NGOs to attend SRH coalition meetings and participate in PAI’s study, in addition to their direct comments, indicated a fear of violating the GGR by merely discussing it. In April 2017, one month before the language implementing the policy was released, a group of organizations that had already chosen noncompliance formed a task force in Addis Ababa to conduct a rapid assessment of how the GGR might impact the health sector. Given that many organizations and professionals had experienced the Bush administration’s more limited GGR on FP/RH, the task force sought to anticipate NGO reactions to the Trump administration’s expansion of the policy to all U.S. global health assistance. The GGR task force found that even early on, organizations were not comfortable discussing the policy or issues related to abortion and were reluctant to provide information about their activities. During the course of PAI’s subsequent research in 2018 and 2019, two non-U.S. NGOs—a prime U.S. global health assistance recipient and a subrecipient—were still unwilling to discuss the GGR out of fear of noncompliance.

Fear of discussing the GGR appeared to be linked to poor communication around the policy’s restrictions and resulting confusion about responsibility for its implementation. At the time of the GGR task force in 2017, the group found that its survey respondents did not have adequate knowledge of the GGR and had not received any communication from either the U.S. government or grant administrators. In February 2018, nearly a year after the April 2017 task force survey, questions from interviewees suggested that confusion and a lack of understanding around the GGR remained, apparent among both non-U.S. organizations and U.S.-based prime recipients. Both PAI and members of the GGR task force provided technical assistance during and after the period of the study. Although U.S. government agencies and prime partners are responsible for policy enforcement, one U.S. organization had not communicated the GGR to its two non-U.S. NGO subrecipients. With the March 2019 interpretation of the policy, there was an added burden on GGR-compliant non-U.S. NGOs, which were required to conduct due diligence on subrecipients of any financial support they provide—regardless of source of funding or activity to be funded. It remained unclear within the Ethiopian SRHR community as to whether certain non-U.S. NGOs, particularly those that would not speak to either PAI or members of the GGR task force, were receiving U.S. funding and if they knew whether they had to comply with the GGR.

## Discussion

Results of the study indicate that the GGR disrupted the health system in Ethiopia by targeting qualified non-U.S. organizations working across the country on safe abortion care. The policy undermined service provision, particularly for adolescents and youth; training on safe abortion care; and partnerships between NGOs and non-U.S. donors. Negative impacts extended well beyond non-U.S. NGOs that chose to not comply with the policy, affecting compliant organizations as well as non-U.S. donors and the public sector. Among these various stakeholders, the GGR disrupted their ability to effectively partner on both funded and non-funded activities. These effects run counter to the explicit SRH policies and goals of the Ethiopian government—including reducing maternal mortality and improving safe abortion care—as well as the U.S. government’s own historical role in improving SRH outcomes in Ethiopia.

Study participants from non-U.S. donors as well as noncompliant and compliant NGOs described how the GGR deprived the most qualified, trusted SRH providers of U.S. funding opportunities to deliver comprehensive services, including safe abortion care, to young people, marginalized groups, and rural populations. Additionally, unlike under the Bush administration—as evidenced by the NGO task force to document the effects of the GGR—organizations were raising awareness of the policy to minimize its over-implementation and counter its harmful impacts. However, at the time of writing, the policy continued to be a source of confusion and fear to the detriment of both compliant and noncompliant NGOs. Regardless of an NGO’s decision to comply, the policy hampered its ability to form effective partnerships—even those without financial dynamics, such as trainings—that advance equitable, nationwide, and comprehensive SRH service delivery beyond safe abortion care.

Findings reveal that mitigating the impact of a policy like the GGR on Ethiopia’s SRHR goals would require coordination among the Ethiopian government, civil society actors, and non-U.S. government donors. Although the Federal Ministry of Health was committed to meeting its FP/RH commitments and indicated support for organizations losing funds because of the GGR, the demand for family planning is immense and the government’s resources are limited. To reinforce this position from the Ethiopian government, some non-U.S. government donors stepped in to fill the gaps for organizations that lost U.S. global health assistance. However, as study participants acknowledged, these non-U.S. donor programs do not reach the same beneficiaries, nor do their funding amounts measure up to the larger U.S. global health assistance opportunities. Because donors have different priorities, objectives, and capacities, one dollar from a non-U.S. government donor is not equivalent to a dollar from a U.S. agency like USAID. Considering this vulnerable environment and the critical role NGOs play in SRH service delivery, advocacy, and technical assistance to the public sector, compliance with the GGR as well as reductions in global health funding for the most qualified, trusted providers that are noncompliant negatively impact the Ethiopian health system—and, ultimately, the health and lives of women, girls, and their communities.

This research on the GGR in Ethiopia was initiated early in the policy’s implementation, meaning a critical limitation was that several non-U.S. NGOs were continuing to close out their U.S. government programs, in the process of finding stopgap funding from non-U.S. government donors, and still determining how compliance or noncompliance would affect their work. As a result, quantifiable loss for activities and beneficiaries was unknown and could have been difficult to determine because of a range of factors, including timing and replacement funding. Additionally, a state of emergency declared in Ethiopia in February 2018 limited the travel capacity of PAI staff to document GGR impacts outside the capital of Addis Ababa. The confusion and fear around the policy also meant that certain organizations receiving U.S. global health assistance were unwilling to be interviewed and, consequently, the impact of the GGR on their activities was unidentified. With the March 2019 financial interpretation of the policy, there may have been additional effects that should be captured as non-U.S. NGOs and non-U.S. government donors subsequently adapted to those changes.

In other countries with progressive liberalized abortion laws where there has been documentation on the effects of the GGR, such as Nepal, the policy has interacted in similar ways, impacting NGO operations—both compliant and noncompliant—and has varied depending on existing national abortion legislation, as well as efforts to liberalize or decriminalize abortion. It is critical to continue documenting the policy’s impact in Ethiopia and globally to understand how SRH impacts may be felt by populations and at the beneficiary level beyond the Trump administration. While the Biden administration rescinded the policy in January 2021, it has not been permanently repealed and could be reinstated.

## Conclusions

This research found that the GGR disrupts SRH service delivery in Ethiopia, with specific implications for safe abortion care, beyond the effects of the policy under previous U.S. administrations. The Trump administration’s expanded GGR fragmented programs, planned activities, and partnerships and forced realignment of government and donor priorities and funding allocations. The policy reached into an organization’s non-U.S. funding and disrupted the priorities and investments of other bilateral and foundation donors, as well as the government of Ethiopia. NGOs are vital for service delivery and can be the only providers for geographically harder-to-reach populations such as rural communities. They are trusted by key populations—such as adolescents and youth, sex workers, and people living with HIV/AIDS—that rely on the private sector for privacy given continued stigma associated with abortion and SRHR broadly. These NGOs depend on funding from donors, like the U.S. government, to provide not only funds but also training and technical support for their programs and partners. Compliance with the policy has implications across the different regions of the country and, as a result, the services available to the population.

Considering the country’s vulnerable SRH funding environment, the reduction in U.S. global health assistance for qualified, trusted NGO providers who refuse to comply with the GGR negatively impacted the Ethiopian health system and potentially the health and lives of women, girls, and community members. As the implementation of one U.S. foreign policy does not occur in a vacuum, GGR impacts were compounded by low domestic resource mobilization for SRH and the uncertainty of continued stopgap funding from non-U.S. government donors. Considering the Ethiopian government’s strong commitments to SRHR, as well as support from other non-U.S. government donors, there is hope that the harmful effects of the GGR will have been partially mitigated—though questions remain at what cost and whether future reinstatement of the policy will be avoided.

## Data Availability

The data that support the findings in this study are available from the corresponding author [JMV] on reasonable request.

## Abbreviations

CBO: Community-based organization
CDC: U.S. Centers for Disease Control and Prevention
FGAE: Family Guidance Association of Ethiopia
FP/RH: Family planning and reproductive health
GGR: Global Gag Rule
NGO: Nongovernmental organization
PEPFAR: U.S. President's Emergency Plan for AIDS Relief
SRH: Sexual and reproductive health
SRHR: Sexual and reproductive health and rights
USAID: U.S. Agency for International Development
